# First Cophylogenetic Reconstruction of Hemipteran Insect–Phytoplasma Associations Reveals Eco-Evolutionary Dynamics

**DOI:** 10.3390/insects17090942

**Published:** 2026-09-09

**Authors:** Valeria Trivellone, Christopher H. Dietrich

**Affiliations:** Illinois Natural History Survey, Prairie Research Institute, University of Illinois at Urbana-Champaign, Champaign, IL 61820, USA; chdietri@illinois.edu

**Keywords:** Auchenorrhyncha, cooccurrence, codivergence, global-fit model, event-based model, *Candidatus* Phytoplasma

## Abstract

Vector-borne plant pathogens exhibit a remarkable capacity to colonize distantly related hosts, reflecting long-term ecological and evolutionary interactions among pathogens, plants, and insect vectors. Phytoplasmas, which are obligate bacterial parasites transmitted by hemipteran insects, represent a model system for investigating how host–pathogen associations diversify across ecological and evolutionary timescales. Despite extensive knowledge of phytoplasma diversity and vector relationships, the historical processes shaping these associations remain poorly understood. Here, we investigated the eco-evolutionary dynamics of global phytoplasma–hemipteran associations using complementary cophylogenetic approaches that integrate phylogenetic relationships, divergence time estimates, and host association data. We tested the hypothesis that community assembly of phytoplasma–vector networks over the last ~300 million years has been driven by multiple processes, including host switching, lineage duplication, and cospeciation, with additional influences from environmental change and historical biogeography. Our analyses revealed limited evidence for cospeciation across the evolution of the associations. Instead, duplication, host switching, and lineage losses were the predominant processes shaping current associations, with more than 90 inferred incongruent links between phytoplasmas and their insect hosts. A significant cophylogenetic signal was detected only between Psyllidae and phytoplasma group 16SrX, suggesting a history of relative ecological stability or specialization following their concurrent emergence in the Late Cretaceous. Reconstruction of the origins of phytoplasma associations with particular families of insect vectors indicates that the common ancestor of modern Cicadellidae may have already acquired an association with phytoplasmas, but that associations with other vector families were acquired more recently. These findings indicate that phytoplasma–vector associations are largely dynamic and shaped by ecological opportunity rather than strict codiversification. Integrating evolutionary history with ecological interactions provides a framework to better understand the emergence, persistence, and potential future spread of vector-borne plant diseases.

## Simple Summary

Analyses comparing phylogenetic trees of phytoplasmas (bacterial plant pathogens) to those of their hemipteran insect vectors show significantly non-random patterns of associations, despite numerous cases of phytoplasmas colonizing multiple vector species, vectors associating with multiple unrelated phytoplasmas and other patterns deviating from strict cospeciation. The results indicate that the ~300-million-year association between phytoplasmas and their insect vectors has been driven by multiple processes (e.g., host switching, losses, failure to diverge, codiversification), some of them being phylogenetically constrained to a certain degree. This provides an eco-evolutionary framework for predicting the potential emergence and spread of vector-borne plant diseases.

## 1. Introduction

Vector-borne plant pathogens retain an extraordinary capability to colonize distantly related organisms at different stages of their often complex life histories, reflecting repeated cycles of host switching, ecological fitting, adaptation, and subsequent establishment [1]. Such pathogens are often associated with, or directly cause, significant economic losses in major agricultural crops, and their interactions with plant and insect hosts require complex modeling approaches to provide a solid theoretical framework for testing hypotheses and generating predictions about future emergence of vector-borne diseases [2,3]. Several authors have suggested that an evolutionary perspective is necessary to understand the mechanisms underlying the expansion and contraction of a pathogen’s host repertoire and to effectively anticipate or predict the risk of emerging diseases, including vector-borne diseases [4,5].

Phytoplasmas are insect-vectored plant pathogens that have undergone extensive reductive evolution, leading to their current status as obligate parasites [6,7,8]. Despite their reduced genomes, they retain key functional genes involved in host (plant and insect) interaction, including effector proteins that modulate host physiology and behavior [9]. These traits may facilitate persistence under changing environmental conditions and host shifts, making phytoplasmas a suitable model for investigating the eco-evolutionary dynamics that shape host–pathogen associations over time. Phytoplasmas are transmitted by hemipteran insects, which acquire the bacteria while feeding on the phloem of infected plants and subsequently inoculate other plants during consecutive feeding events. This repeated exposure of insect populations to phytoplasma cells is considered a prerequisite for the evolution of vector competence. Molecular clock estimates suggest that phytoplasmas and hemipteran insects may have been associated for more than 300 million years, providing substantial evolutionary time for diversification of interactions and transmission strategies [10]. Previous studies have shown that the ability of pathogens to colonize hosts across multiple kingdoms may arise from the evolution of associations that vary along a mutualism–parasitism continuum [11]. These authors proposed a conceptual distinction between carrier hosts and primary hosts, noting that some insect vectors may tolerate infection by certain pathogens, favoring transmission efficiency. In rare cases where insects function both as vectors and primary hosts, such systems may represent transitional stages in the evolution of pathogen–host relationships. More generally, empirical studies in plant pathogen–vector systems support the existence of a broad spectrum of interaction outcomes (from commensal to parasitic) between pathogens and their insect vectors [12]. Such variability is also evident in phytoplasma–vector associations. Some strains have been associated with neutral or positive effects on vector fitness [13], whereas others significantly reduce survival and fecundity [14]. Experimental evidence has also shown that immune responses may differ depending on phytoplasma lineage, as demonstrated in *Euscelidius variegatus* (Kirschbaum), where protective immunity was elicited against one phytoplasma group but not another [15]. Comparable patterns of host-specific adaptation have been observed in other insect–bacterium systems, suggesting that long-term associations may promote efficient retention, transmission, and dispersal [16].

When interacting lineages occupy the same ecological niche over extended periods, they may influence each other in different ways with both ecological and evolutionary consequences. At ecological timescales, processes such as plant-mediated host manipulation can alter transmission dynamics and interaction networks. Over evolutionary timescales, mutual adaptation may lead to co-accommodation [17], whereas concurrent isolation may drive cospeciation [18]. Although concomitant cladogenesis is not necessarily synonymous with coevolution, cophylogenetic analyses provide a widely used framework to investigate the historical assembly of interacting lineages by comparing their phylogenies and identifying signatures of congruence or incongruence [19]. Event-based cophylogenetic approaches further allow the quantification of specific evolutionary events, including cospeciation, host switching, lineage duplication, extinction, and failure to diverge, thereby providing a mechanistic interpretation of host–parasite associations. Integrating phylogenetic relationships with divergence-time estimates and biogeographic information also enables reconstruction of the historical assembly of host–parasite systems and the processes underlying present-day associations [20,21].

Phytoplasmas and their hemipteran insect vectors represent an especially suitable system for such investigations because of their long evolutionary history, obligate transmission, and broad diversity of host associations across lineages [22]. Despite extensive knowledge of phytoplasma diversity and vector relationships, the historical processes that have shaped global phytoplasma–vector associations remain poorly understood. In particular, it remains unclear to what extent these associations reflect long-term codiversification versus repeated host switching and lineage turnover.

In this study we aim to answer three questions: (i) Are current vector–phytoplasma associations phylogenetically structured? (ii) Which evolutionary events best explain those associations? (iii) How did phytoplasma–insect associations evolve through deep time?

We hypothesized that the assembly of phytoplasma–vector associations over the last ~300 million years has been driven primarily by host switching, lineage duplication, and losses rather than strict cospeciation, with additional influences from environmental change and historical biogeography. To test this hypothesis, we applied complementary cophylogenetic reconstruction approaches to a global dataset of phytoplasma–hemipteran associations, integrating phylogenetic information, divergence times, and host association data to infer the dominant processes shaping these interaction networks.

## 2. Materials and Methods

### 2.1. Taxon Sampling and Molecular Markers

Two datasets were considered for the analyses: one containing competent hemipteran vectors and their associated phytoplasma strains (hereafter, the *reduced* dataset), and another containing both competent and putative vectors and their associated phytoplasma strains (hereafter, the *full* dataset). The insect–phytoplasma associations were compiled from published literature and retrieved from the online Hemiptera–Phytoplasma–Plant Biological Interaction Database [23]. The *reduced* dataset includes only competent vectors, defined as species whose ability to acquire phytoplasmas from infected plants and successfully transmit them to another plant has been demonstrated experimentally. Consequently, this dataset represents relatively well-established and well-known vector–phytoplasma associations. In contrast, the *full* dataset also includes the putative vectors, for which vector competence has not been confirmed and whose epidemiological role has not yet been clarified. Here, a hemipteran was classified as a putative vector when evidence from the literature indicated an association with phytoplasmas based on molecular detection (e.g., PCR, qPCR, or sequencing), together with indirect evidence such as repeated association with diseased plants or ecological observations, but without experimental confirmation of phytoplasma transmission. Accordingly, detection of phytoplasma DNA in an insect was considered evidence of an association with the pathogen; however, classification as a putative vector should not be interpreted as evidence of vector competence. Therefore, analyses based on the *full* dataset may include uncertainty arising from unverified or potentially erroneous insect–phytoplasma associations that have not been validated through transmission assays.

Barcode sequences were obtained from the Barcode of Life Data Systems (BOLD) [24] and the National Center for Biotechnology Information (NCBI) database [25]. Molecular data for the *reduced* dataset included 47 hemipteran vector species and 24 phytoplasma strains, and the *full* dataset included 66 hemipteran species and 31 phytoplasma strains (see Supplementary Table S1a,b [26,27,28,29,30,31,32,33,34,35,36,37,38,39,40,41,42,43,44,45,46,47,48,49,50,51]).

Phylogenetic relationships among hemipteran insects were reconstructed using the mitochondrial cytochrome c oxidase subunit I (COI) barcode region. Phylogenetic relationships among phytoplasmas were reconstructed using the *16S ribosomal RNA* (*16S rRNA*) gene, a highly conserved housekeeping marker widely used for bacterial taxonomy and phylogenetic inference (Supplementary Table S1a,b).

### 2.2. Phylogenetic Analyses

Sequences from the *full* and *reduced* datasets were aligned using the MUSCLE algorithm with default settings in MEGA 7.0 [52,53]. The aligned sequences were analyzed in PartitionFinder (v. 2.1.1) to identify the most appropriate substitution models for phylogenetic reconstruction using the corrected Akaike Information Criterion (AICc) [54,55]. For both datasets, the General Time Reversible model with gamma-distributed rate variation among sites (GTR + G) was selected as the best-fitting substitution model based on the lowest AICc value. Phylogenetic trees were inferred using the Maximum Likelihood (ML) method implemented in RAxML [56] using raxmlGUI 2.0 [57]. Branch support was assessed using 1000 bootstrap replicates, with bootstrap support values mapped onto the corresponding branches.

### 2.3. Cophylogenetic Analyses

Phylogenetic trees and the corresponding insect–phytoplasma association matrix were analyzed using two complementary cophylogenetic approaches: global-fit and event-based methods.

Overall phylogenetic congruence between phytoplasma and hemipteran insect phylogenies was assessed using two distance-based global-fit methods: the Procrustean Approach to Cophylogeny (PACo) [58,59], implemented in the R v4.5.0 packages *PACo* v0.4.2 [60] and *vegan* v2.7-2 [61], and ParaFit [62], implemented in the parafit function of the *ape* v5.8-1 R package [63]. Both methods test whether the observed insect–phytoplasma associations exhibit greater phylogenetic congruence than expected by chance and estimate the contribution of individual associations to the overall cophylogenetic signal. Greater congruence is generally interpreted as evidence of a shared evolutionary history, although it may also arise from alternative evolutionary processes, including host switching, duplication (sympatric speciation), and lineage extinction [64].

PACo and ParaFit differ in the hypotheses they evaluate. ParaFit tests whether phytoplasma strains are randomly associated with their hemipteran vectors, whereas PACo evaluates the extent to which phytoplasma phylogeny is constrained by hemipteran phylogeny using a Procrustean goodness-of-fit statistic. Both methods accommodate complex many-to-many association matrices, allowing multiple phytoplasma strains to be associated with multiple vector species and vice versa.

For both analyses, insect and phytoplasma phylogenies were converted to patristic distance matrices using the ape package. ParaFit was performed using the Cailliez correction for negative eigenvalues and 100,000 random permutations of the association matrix to assess the significance of both the global cophylogenetic signal and individual vector–phytoplasma associations. PACo was also run with 100,000 random permutations, and the contribution of individual associations to the global goodness-of-fit statistic was estimated using the jackknife procedure [58,60]. Both analyses were conducted for the *reduced* and *full* datasets. Tanglegrams were generated in R using the *graphics* v4.5.0 package [65].

To complement the distance-based cophylogenetic analyses, we performed an event-based reconciliation analysis using JANE 4.0 [66]. JANE reconstructs the evolutionary history of interacting lineages by identifying the combination of evolutionary events that minimizes the total reconciliation cost between the two phylogenies. Event costs were assigned following four commonly adopted cost schemes in cophylogenetic studies: (0, duplication = 1, host switch (duplication with host switch) = 2, loss = 1, failure to diverge = 1), JANE default values; (0,0,2,1,1), TreeFitter [67] default values; (0,1,1,1,1), TREEMAP [68] version 2b default values; (1,1,1,1,1), equal weights. The optimal reconciliation was inferred using the default genetic algorithm settings (100 generations and a population size of 400). Statistical significance was assessed by comparing the minimum reconciliation cost to a null distribution generated by randomizing insect–phytoplasma tip associations 100 times. A significantly lower observed reconciliation cost than expected under random associations was interpreted as evidence of non-random cophylogenetic congruence between insect vectors and phytoplasma lineages. Event-based reconciliation analyses were conducted only on the *reduced* dataset comprising experimentally confirmed vector–phytoplasma associations, as ancestral event reconstruction requires biologically validated associations.

### 2.4. Ancestral Reconstruction of Phytoplasma Insect Host Repertoire

To investigate the evolutionary history of phytoplasma associations with their insect host families, we reconstructed ancestral vector-family states across the phytoplasma phylogeny using the R-package *corHMM* v2.8 [69]. The analysis was performed using the comprehensive time-calibrated phytoplasma phylogeny by Cao et al. [10], comprising 169 phytoplasma strains. Insect–phytoplasma association data were then compiled from the hemiptera–phytoplasma–plant associations database [23,70]. Both experimentally confirmed vectors and phytoplasma detections in insect bodies were considered as evidence for the association, and the corresponding insect family or superfamily was assigned as the discrete binary character for each analysis. Phytoplasma strains lacking any information on a given insect family were coded as unknown (“?”). The resulting dataset included associations with representatives of one superfamily in the Sternorrhyncha suborder (Psylloidae), and 7 families in the Auchenorrhyncha suborder (Cicadellidae, Delphacidae, Derbidae, Dictyopharidae, Cixiidae, Issidae, Flatidae). Ancestral reconstructions were performed on family-specific pruned phylogenies that retained only phytoplasma strains for which vector associations were known, and separate analyses were conducted for each of the eight major vector families. This approach minimized uncertainty arising from missing observations while preserving the original branch lengths and divergence times of the dated phylogeny. Ancestral states were reconstructed under continuous-time Markov models implemented in corHMM. Model comparison between the Equal Rates (ER) and All Rates Different (ARD) models was performed for each vector family using AICc (Supplementary Table S2). Because the ARD model provided the best fit for seven of the eight vector families, ancestral-state reconstructions were based on the ARD model. The ER and ARD models nevertheless produced nearly identical ancestral-state probabilities, inferred acquisition ages, and numbers of independent acquisition events. Marginal ancestral-state probabilities were estimated for every internal node.

To quantify phylogenetic conservatism of each insect-associated family, the D statistic by Fritz and Purvis [71] was calculated using the R-package *caper* v1.0.4 [72], comparing the observed distribution of vector associations against expectations under random and Brownian models of trait evolution. Values of D near 1 indicate phylogenetically random distributions, whereas values approaching 0 indicate Brownian phylogenetic structure. Negative values indicate stronger phylogenetic clustering than expected under Brownian evolution.

To infer the temporal origin of vector-family associations, ancestral-state probabilities were combined with node ages from the time-calibrated phylogeny. For vector families reconstructed as absent at the root but present within descendant clades, independent evolutionary origins were identified as transitions from low ancestral probability (*p* < 0.5) to high descendant probability (*p* > 0.9). The age of each acquisition event was estimated as the midpoint between the parent and descendant node ages. For families reconstructed as already associated near the root of the phytoplasma phylogeny, the association was interpreted as predating the diversification of extant phytoplasma lineages, and no internal acquisition age was estimated.

## 3. Results

### 3.1. Phylogenetic Analysis

The hemipteran dataset comprised species from five families, including three families within the suborder Auchenorrhyncha (Cicadellidae, Cixiidae, Dictyopharidae) and two families within Sternorrhyncha (Psyllidae and Triozidae). The insect phylogenies for the two datasets were reconstructed from alignments of length 658 and 663 bp, respectively. The resulting maximum-likelihood trees recovered the major taxonomic groups with strong support and were largely consistent with current classifications of leafhoppers, planthoppers, psyllids, spittlebugs, and related hemipteran lineages [73]. The phytoplasma phylogeny was reconstructed from alignments of representative 16Sr subgroup sequences for the *reduced* and *full* datasets spanning 1463 and 1518 bp of the *16S rRNA* gene, respectively. The phylogenies recovered the recognized relationships among phytoplasma groups, with closely related 16Sr groups clustering together [74].

To evaluate cophylogenetic congruence, these independently reconstructed phylogenies were subsequently linked through experimentally confirmed vector–phytoplasma associations (*reduced* dataset) and through the *full* dataset including putative associations (Section 3.2).

### 3.2. Cophylogenetic Analysis

**Figure 1:**
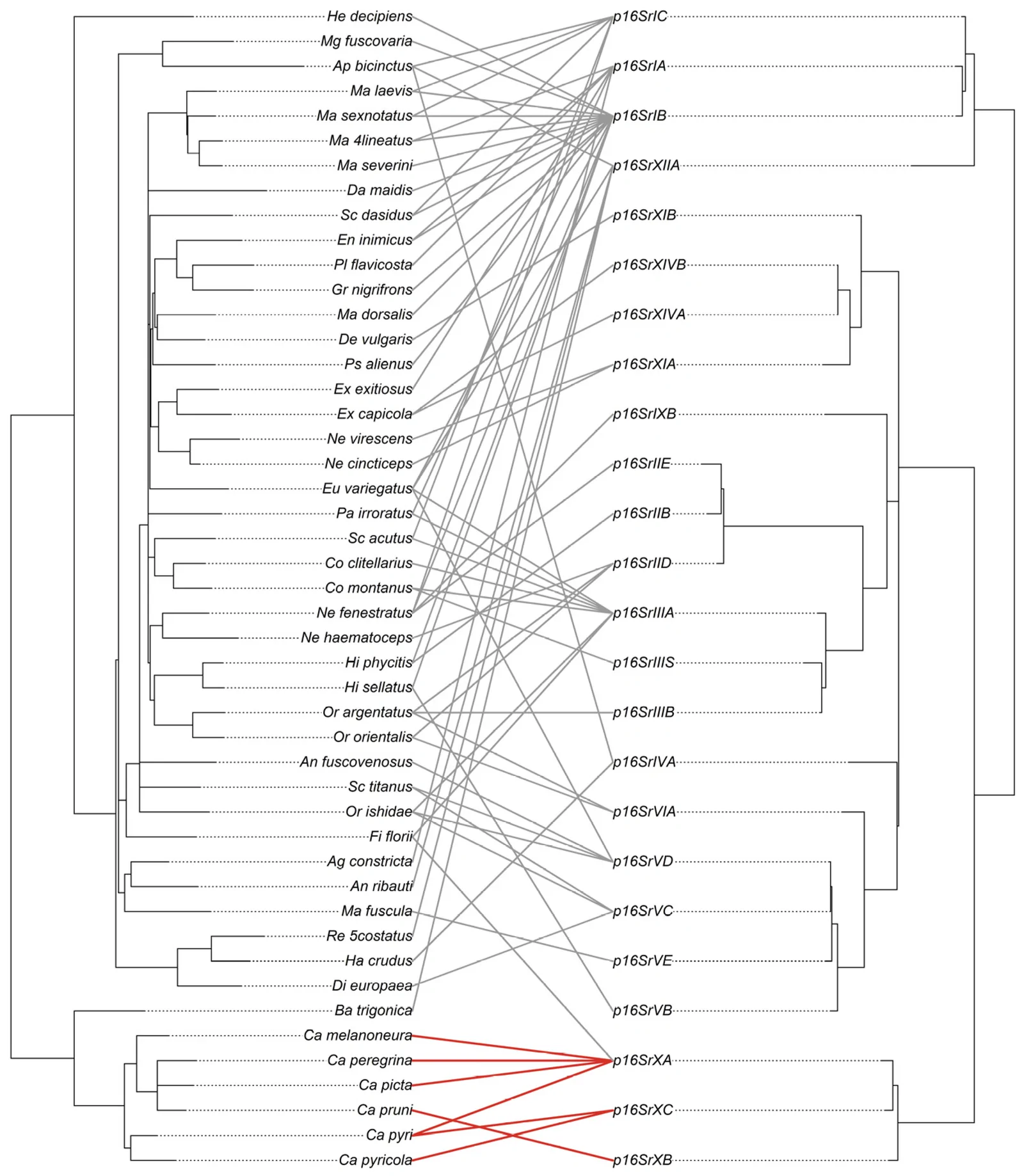
Tanglegram illustrating the cophylogenetic relationships between hemipteran vectors (**left**) and phytoplasma 16Sr subgroups (**right**) based on the *reduced* dataset containing only experimentally confirmed vector–phytoplasma associations. Red lines highlight associations involving species of *Cacopsylla*, which collectively accounted for the strongest cophylogenetic signal in both PACo and ParaFit analyses. The topology summarizes the maximum-likelihood phylogenies reconstructed independently for insects and phytoplasmas.

The *reduced* dataset comprised 47 hemipteran vector species and 24 phytoplasma strains connected by 74 experimentally confirmed vector–phytoplasma associations. The *full* dataset comprised 66 hemipteran species and 31 phytoplasma strains connected by 135 associations, including both experimentally confirmed and putative interactions. The overall pattern of associations in the *reduced* dataset is illustrated by the cophylogenetic tanglegram (Figure 1), whereas the corresponding tanglegram for the *full* dataset is provided in Supplementary Figure S1.

Both PACo and ParaFit recovered significant overall cophylogenetic congruence between hemipteran vectors and phytoplasma lineages. PACo yielded a global goodness-of-fit of m^2^ = 0.81 (*p* value < 0.001) for the *reduced* dataset and m^2^ = 0.84 (*p* value < 0.001) for the *full* dataset. Likewise, ParaFit detected significant global congruence for both the *reduced* dataset (ParaFitGlobal = 2.54, *p* = 0.001) and the *full* dataset (ParaFitGlobal = 8.65, *p* = 0.001) (Table 1).

Jackknife residuals from PACo revealed substantial heterogeneity among individual associations (Supplementary Table S3a,b). In the *reduced* dataset, the largest residuals were concentrated within the *Cacopsylla*-16SrX associations, including *C. peregrina*-16SrXA, *C. pictai*-16SrXA, *C. pruni*-16SrXB, *C. pyricola*-16SrXC, *C. melanoneura*-16SrXA, and *C. pyri*-16SrXA and XC. All of these associations were individually significant according to ParaFit. Similar results were obtained for the *full* dataset, where the same *Cacopsylla*-16SrX associations remained among the highest-ranking and were consistently supported by ParaFit despite the inclusion of putative vector–phytoplasma associations. The *full* dataset additionally recovered several individually significant associations with comparatively lower PACo residuals, including *Nephotettix virescens*-16SrXIA, *Exitianus capicola*-16SrXIVA/XIVB, and *Orosius orientalis*-16SrIIC/IID/IIE. Comparison of individual associations showed broad agreement between PACo and ParaFit in both datasets (Supplementary Table S3a,b). Nearly all of the high-ranking associations identified by PACo were also individually significant in ParaFit, particularly those involving the *Cacopsylla*-16SrX complex. Across all interactions, PACo jackknife residuals and ParaFit F1 statistics were positively correlated in both the *reduced* (Pearson’s *r* = 0.54) and *full* (Pearson’s *r* = 0.50) datasets, indicating broad agreement between the two methods in identifying evolutionarily important insect–phytoplasma associations.

Event-based reconciliation analyses performed in JANE produced slightly different combinations of evolutionary events depending on the cost scheme employed (Table 2), consistent with previous studies showing that reconciliation outcomes are sensitive to assumptions regarding event costs [75]. Nevertheless, all four cost schemes recovered highly significant cophylogenetic structure (*p* = 0.001) and converged on the same overall evolutionary scenario, in which losses, host-switching events, and failure-to-diverge events greatly outnumbered cospeciation events. Importantly, this pattern remained unchanged even under reconciliation schemes that penalized host switching more heavily, indicating that the inferred evolutionary history is robust to reasonable variation in event costs. Across the four cost schemes, the optimal reconciliation inferred one or two cospeciation events, 9–12 duplications, 9–13 host switch events, 118–123 losses, and 50 failure-to-diverge events (Table 2). Losses were consistently the most frequent reconciliation event, followed by failure-to-diverge events, whereas cospeciation was rare. Notably, event-based reconciliation did not infer cospeciation for the *Cacopsylla*-16SrX complex. Instead, these associations were primarily reconstructed as duplication and host-switching events; their evolutionary history was explained by an ancestral duplication followed by host switching and subsequent diversification within the *Cacopsylla* lineage. Conversely, the few inferred cospeciation events involved associations that also exhibited some of the lowest PACo residuals, including the *Nephotettix*-16SrXI-A association, indicating that these represent the strongest candidates for genuine codiversification.

### 3.3. Evolutionary History of Phytoplasma–Insect Family Associations

Ancestral-state reconstructions revealed marked differences in the evolutionary history of phytoplasma associations among the major families of insect vectors (Table 3). Cicadellidae showed the strongest evidence of long-term association with phytoplasmas. The marginal probability of presence at the root node was 0.79, providing strong support for an ancestral association between phytoplasmas and Cicadellidae. Posterior probabilities remained close to one across the deepest internal nodes, suggesting that this association was likely already established in the common ancestor of extant phytoplasmas (~300 Ma). This interpretation was further supported by the significant phylogenetic conservatism of Cicadellidae associations (D = 0.47, P *random* = 0.003; P *Brownian* = 0.035).

In contrast, Psylloidea showed a low root probability (*p* = 0.16), indicating that associations with this vector family were acquired after the diversification of the phytoplasma ancestral lineage. A single acquisition of phytoplasma association with this superfamily was inferred at 176.6 million years ago (Ma), followed by diversification within the 16SrX lineage. Likewise, Cixiidae displayed a low ancestral probability at the root (*p* = 0.24), but two independent acquisition events were inferred, occurring at approximately 40.5 Ma and 24.5 Ma, suggesting repeated recruitment of this vector lineage during phytoplasma evolution.

The remaining vector families (Derbidae, Dictyopharidae, Delphacidae, Flatidae, and Issidae) exhibited very low root probabilities (<0.05), also indicating relatively recently derived associations restricted to relatively few phytoplasma lineages. Several vector families showed strong phylogenetic clustering (negative D values), indicating that their associations are concentrated within particular phytoplasma lineages (e.g., Psylloidea and 16SrX). In contrast, families with positive D values exhibited weaker phylogenetic structure, suggesting broader evolutionary distributions across the phytoplasma phylogeny.

## 4. Discussion

As previously noted by Nadarasah and Stavrinides [11], the ability of some pathogens to colonize hosts from more than one kingdom may arise from the evolution of associations spanning the mutualism–parasitism continuum. These authors also proposed a distinction between ‘carriers’ and ‘primary hosts’, referring to whether the pathogen causes pathology in the insect vector (i.e., its entomopathogenic potential). They argued that vector efficiency may depend on evolutionary trade-offs that reduce pathogenic effects while maintaining transmission. Because only rare cases are known in which an insect acts simultaneously as both vector and primary host, this condition has been interpreted as a possible transitional stage in the evolution of phytopathogen–vector and phytopathogen–host associations. Our cophylogenetic analyses provide an evolutionary framework for evaluating how these long-term associations originated and diversified across phytoplasma lineages.

Restricting our analyses to experimentally confirmed vector–phytoplasma associations strengthened the cophylogenetic signal and increased the agreement between PACo and ParaFit. This suggests that uncertain or unverified associations may introduce noise that obscures the understanding of the underlying evolutionary relationships. The strongest concordance between insect–phytoplasma lineages, and for the two algorithms, was observed for the *Cacopsylla*-16SrX associations, supporting the hypothesis that these interactions are evolutionarily conserved. In contrast, *Aphrodes bicinctus* (now *A. bicincta*) consistently ranked among the strongest contributors in PACo but was not individually significant in ParaFit. Because PACo measures the contribution of each interaction to the overall Procrustes fit whereas ParaFit assesses whether a particular interaction contributes uniquely to the observed cophylogenetic pattern, this discrepancy likely reflects the relatively generalist ecology of *A. bicincta*, whose multiple phytoplasma associations reinforce the overall phylogenetic structure without being individually indispensable. We are aware that PACo tests the classical view of whether parasite phylogeny is constrained by the host phylogeny, and does not explicitly test evolutionary scenarios [76]. Congruence alone should not be interpreted as evidence of codiversification. Distance-based methods quantify the degree of phylogenetic correspondence between hosts and pathogens but do not identify the evolutionary processes generating that correspondence. Consequently, the significant global congruence recovered by PACo and ParaFit provides evidence for non-random evolutionary associations but does not distinguish between cospeciation, host switching, duplication, or lineage persistence.

Although the vector–phytoplasma association network exhibited significant cophylogenetic structure, event-based reconciliation analysis suggested that this congruence has not been generated primarily through strict codiversification. Across all reconciliation cost schemes, losses and failure-to-diverge events overwhelmingly dominated the inferred evolutionary history, whereas cospeciation events were rare. Event-based reconciliation therefore suggests that the significant phylogenetic congruence detected by PACo and ParaFit did not arise primarily through repeated parallel diversification of phytoplasmas and their insect vectors. Instead, historical host switches may have established associations with particular vector lineages, and these associations subsequently may have persisted and diversified within those lineages, producing phylogenetic congruence that resembles codiversification even though true cospeciation events were rare. Losses occur when a host lineage diversifies, but the pathogen persists in only one descendant host lineage, whereas failure to diverge occurs when host speciation is not accompanied by pathogen speciation and the same pathogen lineage continues to infect multiple descendant host species [66]. This interpretation is consistent with the concept of ecological fitting, whereby pathogens exploit conserved ecological or physiological traits shared among related hosts without requiring substantial evolutionary innovation [77]. Remarkably, the *Cacopsylla*-16SrX associations that were key contributors to the global cophylogenetic signal were not recovered in the event-based approach as the primary locations of cospeciation events, but were instead explained by an ancestral duplication and host switch followed by diversification within the *Cacopsylla* lineage. This result provides a concrete example of the broader evolutionary scenario inferred by the reconciliation analyses: the strong phylogenetic congruence observed for the 16SrX phytoplasma group and its psyllid vectors appears to have arisen through an ancestral host switch followed by diversification within the newly colonized vector lineage rather than through repeated cospeciation. Similar evolutionary scenarios have recently been proposed for other vector-borne and plant-associated pathogens. For example, studies of the xylem-borne bacterial plant pathogen *Xylella fastidiosa* demonstrated that host specialization arose repeatedly following historical host switches across distantly related plant lineages rather than through gradual diversification along host phylogenies [78]. Likewise, analyses of Sclerotiniaceae fungi concluded that broad host ranges evolved primarily through repeated host switches combined with relatively low pathogen speciation rates. Together, these studies support the emerging view that host shifts, followed by ecological specialization within the newly colonized lineage, represent a common mechanism underlying host-associated pathogen diversification [79].

Our ancestral-state reconstructions further support this scenario by revealing marked differences in the evolutionary history of phytoplasma associations with individual families of insect vectors. Cicadellidae exhibited the strongest phylogenetic conservatism (D ~ 0.47), with ancestral-state reconstruction indicating a high probability that this association was already present at the root of the phytoplasma phylogeny. This suggests that leafhoppers represent the ancestral vector lineage and that their association predates the diversification of extant phytoplasmas. In contrast, Cixiidae displayed a more recent origin, with two independent acquisitions estimated at approximately 41 Ma and 25 Ma, indicating repeated recruitment of vectors in this planthopper family during phytoplasma evolution. Psylloidea presented an intermediate pattern. Despite an extremely clustered distribution (D < 0), ancestral reconstruction suggested a single deep acquisition near the base of the 16SrX lineage followed by long-term evolutionary persistence. This timing is broadly compatible with estimates for the radiation of both 16SrX phytoplasmas (approximately 96 Ma [10]) and Psylloidea (approx. 100–338 Ma [73], even though Wu et al. [80] estimated a much more recent time of origin, 49–129 Ma, based on a limited outgroup sample), supporting the hypothesis that this highly specialized association originated early in the diversification of both groups. Whether this pattern reflects an ancient host switch followed by geographic isolation or an ancestral association subsequently lost from all other phytoplasma lineages remains unresolved, but both scenarios are considerably more parsimonious than repeated independent acquisitions.

This evolutionary framework reconciles the apparent contradiction between significant cophylogenetic signal and the predominance of host-switching events, suggesting that present-day phytoplasma–vector specificity reflects the long-term stabilization of successful host colonization events rather than continuous parallel diversification of pathogens and their insect vectors. These results are consistent with previous studies that suggested that long-term plant-pathogen cospeciation is rare [18].

All the well-documented phytoplasma–vector associations are based on research in agroecosystems or anthropogenic environments, which have shown global losses in biodiversity due to continuous disturbance [81]. As a consequence, phytoplasma–host associations may not be representative of the broader diversity of such associations in nature [22]. Recent research suggests that a large proportion of extant phytoplasma diversity remains undiscovered in natural areas and the numbers and identities of potential vectors are also largely unknown [82,83,84]. This clearly represents a limitation for our study, and the cophylogenetic patterns revealed here will need to be revisited as the currently undocumented diversity of these associations becomes better known.

A second consideration concerns the evolutionary history of the vectors themselves. Phylogenetic relationships among the major leafhopper lineages remain incompletely resolved, probably because an ancient rapid radiation gave rise to most extant cicadellid subfamilies over a relatively short evolutionary interval during the Mesozoic, as reflected by the short internal branches connecting the principal lineages [85]. This uncertainty is unlikely to have substantially affected the present analyses because the currently confirmed phytoplasma vectors belong to only six of the nineteen recognized cicadellid subfamilies and most are associated with a single subfamily, Deltocephalinae. Nevertheless, future studies incorporating newly discovered phytoplasma–insect associations across a broader taxonomic spectrum should consider the potential influence of uncertainty in vector phylogeny on cophylogenetic inference and ancestral-state reconstruction.

## 5. Conclusions

Taken together, these results indicate that phytoplasma diversification has been shaped by a combination of ancient, evolutionarily stable vector associations and more recent episodes of vector-family recruitment. Rather than a history dominated by strict codiversification, phytoplasma evolution appears to follow a dynamic process in which occasional host switches establish novel vector associations that subsequently become evolutionarily conserved within particular lineages. Once established, these associations persist through failure-to-diverge events and repeated transmission among closely related vector species, producing the strong phylogenetic signal detected by distance-based methods despite relatively few true cospeciation events.

## Figures and Tables

**Table 1:** Outcome of the cophylogenetic analyses in PACo and ParaFit for the *reduced* and *full* datasets.

Datasets	No. Links	PACo		ParaFit			
		m^2^	*p* Value	F1.stat	*p*.F1	No. Sign. Links	% Sign. Links/Tot
Reduced ^1^	74	0.81	<0.00001	2.54	0.001	7	9.5
Full ^2^	135	0.84	<0.00001	8.65	0.001	28	20.7

^1^ Including 47 confirmed hemipteran vectors and 24 associated-phytoplasma strains. ^2^ Including 66 confirmed and potential hemipteran vectors and 31 associated-phytoplasma strains.

**Table 2:** Outcome of the cophylogenetic analyses in JANE, employing various cost schemes. The number of different event types retrieved and the statistical significance based on 1000 randomizations are indicated for each reconstruction. Event costs are ordered as codivergence, duplication, host switch, loss, and failure to diverge.

Event Cost	Codivergence	Duplication	Host Switch	Loss	Failure to Diverge	Total Cost	*p* Value
(0,1,2,1,1) ^1^	2	9	12	119	50	202	0.001
(0,0,2,1,1) ^2^	2	12	9	123	50	191	0.001
(0,1,1,1,1) ^3^	1	9	13	118	50	190	0.001
(1,1,1,1,1) ^4^	1	9	13	118	50	191	0.001

^1^ JANE default values; ^2^ TreeFitter default values; ^3^ TREEMAP version 2b default values; ^4^ Equal weights.

**Table 3:** Phylogenetic conservatism and ancestral reconstruction of major phytoplasma vector families. For each insect family/superfamily, the table reports the marginal probability that the association was present at the root of the phytoplasma phylogeny (Root prob.), phylogenetic D statistic, and the associated probabilities that the observed distribution does not differ from a random distribution (P(r)) or from the expectation under Brownian evolution (P(Br)). Values of D near 1 indicate a random phylogenetic distribution, values near 0 indicate Brownian phylogenetic structure, and negative values indicate stronger phylogenetic clustering than expected under Brownian evolution. The last two columns report the estimated age (million years ago, Ma) of the earliest inferred acquisition of each vector family (Ass. Ma) and inferred number of independent acquisition events (# origins).

Family/ Superfamily	Root Prob.	D Statistic	P(r)	P(Br)	Ass (Ma)	# Origins
Cicadellidae	0.79	0.47	0.003	0.035	Near crown	Ancestral
Psylloidea	0.20	−0.58	<0.001	0.889	176.6	1
Cixiidae	0.18	0.26	0.001	0.204	40.5	2
Derbidae	0.04	1.21	0.647	0.056	-	0
Dictyopharidae	0.06	0.73	0.168	0.108	-	0
Delphacidae	0.05	0.45	0.114	0.386	-	0
Flatidae	0.03	−3.82	0.069	0.824	-	0
Issidae	0.02	−3.11	0.151	0.709	-	0

## Data Availability

The original contributions presented in this study are included in the article/Supplementary Materials. Further inquiries can be directed to the corresponding author.

## Supplementary Materials

The following supporting information can be downloaded at https://www.mdpi.com/article/10.3390/insects17090942/s1, Table S1a: List of cytochrome c oxidase subunit I (COI) gene sequences downloaded from GenBank or BOLD. na: not assigned. * = hemipteran species selected for the reduced dataset which includes known competent vectors; Table S1b: List of *16S rRNA* gene sequences downloaded from GenBank. na: data not available. * = strain selected for the reduced dataset which includes phytoplasmas for which competent vector species is (are) known; Table S2. Comparison of Equal Rates (ER) and All Rates Different (ARD) models used for ancestral-state reconstruction; Table S3a: Individual insect host–phytoplasma associations included in the reduced dataset used for cophylogenetic analyses. ParaFitLink statistics (F1.stat and F2.stat), permutation-based *p* values (*p*.F1 and *p*.F2), PACo residuals, and the inferred support for each interaction are shown. Significant ParaFitLink tests indicate associations contributing significantly to the global cophylogenetic pattern, whereas lower PACo residuals denote stronger contributions to the overall Procrustes fit; Table S3b: Individual insect host–phytoplasma associations included in the full dataset used for cophylogenetic analyses. ParaFitLink statistics (F1.stat and F2.stat), permutation-based *p* values (*p*.F1 and *p*.F2), PACo residuals, and the inferred support for each interaction are shown. Significant ParaFitLink tests indicate associations contributing significantly to the global cophylogenetic pattern, whereas lower PACo residuals denote stronger contributions to the overall Procrustes fit; Figure S1: Tanglegram of the full dataset including experimentally confirmed vector–phytoplasma associations together with putative associations reported from phytoplasma detections in insects. Red lines highlight associations involving insect species which collectively accounted for the strongest cophylogenetic signal in both PACo and ParaFit analyses. The topology summarizes the maximum-likelihood phylogenies reconstructed independently for insects and phytoplasmas.

## References

[1] Huang Y., Hao J., Tang X. (2025). Bacterial Vector-Borne Plant Diseases: Global Issues Caused by Three-Way Interactions. Crop Health.

[2] Huang W., Reyes-Caldas P., Mann M., Seifbarghi S., Kahn A., Almeida R.P.P., Béven L., Heck M., Hogenhout S.A., Coaker G. (2020). Bacterial Vector-Borne Plant Diseases: Unanswered Questions and Future Directions. Mol. Plant.

[3] Trivellone V., Araujo S., Dietrich C.H. (2025). Evolutionary Insights into Hemipteran Insect-Phytoplasma Associations: Phylogenetic Conservatism and Historical Ecology as Tools to Predict Future Associations. Phytoplasmas: Genomes, Plant Hosts and Vectors.

[4] Thines M. (2019). An Evolutionary Framework for Host Shifts—Jumping Ships for Survival. New Phytol..

[5] Gardner S.L., Brooks D.R., Boeger W.A., Hoberg E.P. (2023). An Evolutionary Pathway for Coping with Emerging Infectious Disease.

[6] Kube M., Mitrovic J., Duduk B., Rabus R., Seemüller E. (2012). Current View on Phytoplasma Genomes and Encoded Metabolism. Sci. World J..

[7] Oshima K., Kakizawa S., Nishigawa H., Jung H.-Y., Wei W., Suzuki S., Arashida R., Nakata D., Miyata S., Ugaki M. (2004). Reductive Evolution Suggested from the Complete Genome Sequence of a Plant-Pathogenic Phytoplasma. Nat. Genet..

[8] Oshima K., Maejima K., Namba S. (2013). Genomic and Evolutionary Aspects of Phytoplasmas. Front. Microbiol..

[9] Orlovskis Z., Hogenhout S.A. (2016). A Bacterial Parasite Effector Mediates Insect Vector Attraction in Host Plants Independently of Developmental Changes. Front. Plant Sci..

[10] Cao Y., Trivellone V., Dietrich C.H. (2020). A Timetree for Phytoplasmas (Mollicutes) with New Insights on Patterns of Evolution and Diversification. Mol. Phylogenet. Evol..

[11] Nadarasah G., Stavrinides J. (2011). Insects as Alternative Hosts for Phytopathogenic Bacteria. FEMS Microbiol. Rev..

[12] Stavrinides J., No A., Ochman H. (2010). A Single Genetic Locus in the Phytopathogen *Pantoea stewartii* Enables Gut Colonization and Pathogenicity in an Insect Host. Environ. Microbiol..

[13] Orlovskis Z., Singh A., Kliot A., Huang W., Hogenhout S.A. (2025). The Phytoplasma SAP54 Effector Acts as a Molecular Matchmaker for Leafhopper Vectors by Targeting Plant MADS-Box Factor SVP. eLife.

[14] Bressan A., Clair D., Sémétey O., Boudon-Padieu É. (2005). Effect of Two Strains of Flavescence Dorée Phytoplasma on the Survival and Fecundity of the Experimental Leafhopper Vector *Euscelidius variegatus* Kirschbaum. J. Invertebr. Pathol..

[15] Galetto L., Abbà S., Rossi M., Vallino M., Pesando M., Arricau-Bouvery N., Dubrana M.-P., Chitarra W., Pegoraro M., Bosco D. (2018). Two Phytoplasmas Elicit Different Responses in the Insect Vector *Euscelidius variegatus* Kirschbaum. Infect. Immun..

[16] Quevillon-Cheruel S., Leulliot N., Muniz C.A., Vincent M., Gallay J., Argentini M., Cornu D., Boccard F., Lemaître B., van Tilbeurgh H. (2009). Evf, a Virulence Factor Produced by the *Drosophila* Pathogen *Erwinia carotovora*, Is an S-Palmitoylated Protein with a New Fold That Binds to Lipid Vesicles. J. Biol. Chem..

[17] Brooks D.R. (1979). Testing the Context and Extent of Host-Parasite Coevolution. Syst. Biol..

[18] Vienne D.M.d.e., Refrégier G., López-Villavicencio M., Tellier A., Hood M.E., Giraud T. (2013). Cospeciation vs Host-Shift Speciation: Methods for Testing, Evidence from Natural Associations and Relation to Coevolution. New Phytol..

[19] Dismukes W., Braga M.P., Hembry D.H., Heath T.A., Landis M.J. (2022). Cophylogenetic Methods to Untangle the Evolutionary History of Ecological Interactions. Annu. Rev. Ecol. Evol. Syst..

[20] Brooks D.R. (1985). Historical Ecology: A New Approach to Studying the Evolution of Ecological Associations. Ann. Mo. Bot. Gard..

[21] Page R.D.M. (2003). Tangled Trees: Phylogeny, Cospeciation, and Coevolution.

[22] Trivellone V., Dietrich C.H. (2021). Evolutionary Diversification in Insect Vector-Phytoplasma-Plant Associations. Ann. Entomol. Soc. Am..

[23] Trivellone V. (2019). An Online Global Database of *Hemiptera*-Phytoplasma-Plant Biological Interactions. Biodivers. Data J..

[24] Ratnasingham S., Herbert P.D.N. (2007). Bold: The Barcode of Life Data System (http://www.barcodinglife.org). Mol. Ecol. Notes.

[25] Federhen S. (2012). The NCBI Taxonomy Database. Nucleic Acids Res..

[26] Arocha Y., López M., Piñol B., Fernández M., Picornell B., Almeida R., Palenzuela I., Wilson M.R., Jones P. (2005). “Candidatus Phytoplasma Graminis” and “Candidatus Phytoplasma Caricae”, Two Novel Phytoplasmas Associated with Diseases of Sugarcane, Weeds and Papaya in Cuba. Int. J. Syst. Evol. Microbiol..

[27] Arocha-Rosete Y., Zunnoon-Khan S., Krukovets I., Crosby W., Scott J., Bertaccini A., Michelutti R. (2011). Identification and Molecular Characterization of the Phytoplasma Associated with Peach Rosette-like Disease at the Canadian Clonal Genebank Based on the 16S rRNA Gene Analysis. Can. J. Plant Pathol..

[28] Davis R.E., Dally E.L. (2001). Revised Subgroup Classification of Group 16SrV Phytoplasmas and Placement of Flavescence Dorée-Associated Phytoplasmas in Two Distinct Subgroups. Plant Dis..

[29] Davis R.E., Harrison N.A., Zhao Y., Wei W., Dally E.L. (2016). “Candidatus Phytoplasma Hispanicum”, a Novel Taxon Associated with Mexican Periwinkle Virescence Disease of Catharanthus Roseus. Int. J. Syst. Evol. Microbiol..

[30] Davis R.E., Jomantiene R., Kalvelyte A., Dally E.L. (2003). Differential Amplification of Sequence Heterogeneous Ribosomal RNA Genes and Classification of the “Fragaria Multicipita” Phytoplasma. Microbiol. Res..

[31] Griffiths H.M., Sinclair W.A., Smart C.D., Davis R.E. (1999). The Phytoplasma Associated with Ash Yellows and Lilac Witches’-Broom: “Candidatus Phytoplasma Fraxini”. Int. J. Syst. Bacteriol..

[32] Hiruki C., Wang K. (2004). Clover Proliferation Phytoplasma: “Candidatus Phytoplasma Trifolii.”. Int. J. Syst. Evol. Microbiol..

[33] Jung H.-Y., Sawayanagi T., Wongkaew P., Kakizawa S., Nishigawa H., Wei W., Oshima K., Miyata S.-I., Ugaki M., Hibi T. (2003). “Candidatus Phytoplasma Oryzae”, a Novel Phytoplasma Taxon Associated with Rice Yellow Dwarf Disease. Int. J. Syst. Evol. Microbiol..

[34] Lee I.-M., Bottner-Parker K.D., Zhao Y., Davis R.E., Harrison N.A. (2010). Phylogenetic Analysis and Delineation of Phytoplasmas Based on secY Gene Sequences. Int. J. Syst. Evol. Microbiol..

[35] Lee I.-M., Gundersen-Rindal D.E., Davis R.E., Bottner K.D., Marcone C., Seemüller E. (2004). “Candidatus Phytoplasma Asteris”, a Novel Phytoplasma Taxon Associated with Aster Yellows and Related Diseases. Int. J. Syst. Evol. Microbiol..

[36] Lee I.-M., Martini M., Marcone C., Zhu S.F. (2004). Classification of Phytoplasma Strains in the Elm Yellows Group (16SrV) and Proposal of “Candidatus Phytoplasma Ulmi” for the Phytoplasma Associated with Elm Yellows. Int. J. Syst. Evol. Microbiol..

[37] Malembic-Maher S., Salar P., Filippin L., Carle P., Angelini E., Foissac X. (2011). Genetic Diversity of European Phytoplasmas of the 16SrV Taxonomic Group and Proposal of “Candidatus Phytoplasma Rubi”. Int. J. Syst. Evol. Microbiol..

[38] Marcone C., Gibb K.S., Streten C., Schneider B. (2004). “Candidatus Phytoplasma Spartii”, “Candidatus Phytoplasma Rhamni” and “Candidatus Phytoplasma Allocasuarinae”, Respectively Associated with Spartium Witches’-Broom, Buckthorn Witches’-Broom and Allocasuarina Yellows Diseases. Int. J. Syst. Evol. Microbiol..

[39] Salehi M., Izadpanah K., Siampour M., Taghizadeh M. (2009). Molecular Characterization and Transmission of Bermuda Grass White Leaf Phytoplasma in Iran. J. Plant Pathol..

[40] Santos-Cervantes M.E., Chávez-Medina J.A., Acosta-Pardini J., Flores-Zamora G.L., Méndez-Lozano J., Leyva-López N.E. (2010). Genetic Diversity and Geographical Distribution of Phytoplasmas Associated with Potato Purple Top Disease in Mexico. Plant Dis..

[41] Schneider B., Cousin M.T., Klinkong S., Seemüller E. (1995). Taxonomic Relatedness and Phylogenetic Positions of Phytoplasmas Associated with Diseases of Faba Bean, Sunnhemp, Sesame, Soybean, and Eggplant/Taxonomische Verwandtschaft Und Phylogenetische Stellung von Phytoplasmen, Die Krankheiten Bei Pferdebohne, Crotalaria, Sesam, Sojabohne Und Aubergine Hervorrufen. J. Plant Dis. Prot..

[42] Seemüller E., Schneider B., Mäurer R., Ahrens U., Daire X., Kison H., Lorenz K.H., Firrao G., Avinent L., Sears B.B. (1994). Phylogenetic Classification of Phytopathogenic Mollicutes by Sequence Analysis of 16S Ribosomal DNA. Int. J. Syst. Bacteriol..

[43] Seemüller E., Schneider B. (2004). “Candidatus Phytoplasma Mali”, “Candidatus Phytoplasma Pyri” and “Candidatus Phytoplasma Prunorum”, the Causal Agents of Apple Proliferation, Pear Decline and European Stone Fruit Yellows, Respectively. Int. J. Syst. Evol. Microbiol..

[44] Seemüller E., Marcone C., Lauer U., Ragozzino A., Göschl M. (1998). Current Status of Molecular Classification of the Phytoplasmas. J. Plant Pathol..

[45] Seruga M., Skoric D., Botti S., Paltrinieri S., Juretic N., Bertaccini A.F. (2003). Molecular Characterization of a Phytoplasma from the Aster Yellows (16SrI) Group Naturally Infecting *Populus nigra* L. “Italica” Trees in Croatia. For. Pathol..

[46] Torres E., Botti S., Rahola J., Martín M.P., Bertaccini A. (2005). Grapevine Yellows Diseases in Spain: Eight Year Survey of Disease Spread and Molecular Characterization of Phytoplasmas Involved. An. Jard. Bot. Madr..

[47] Tymon A.M., Jones P., Harrison N.A. (1998). Phylogenetic Relationships of Coconut Phytoplasmas and the Development of Specific Oligonucleotide PCR Primers. Ann. Appl. Biol..

[48] Verdin E., Salar P., Danet J.-L., Choueiri E., Jreijiri F., El Zammar S., Gélie B., Bové J.M., Garnier M. (2003). “Candidatus Phytoplasma Phoenicium” sp. Nov., a Novel Phytoplasma Associated with an Emerging Lethal Disease of Almond Trees in Lebanon and Iran. Int. J. Syst. Evol. Microbiol..

[49] Wang J., Liu Q., Wei W., Davis R.E., Tan Y., Lee I.-M., Zhu D., Wei H., Zhao Y. (2018). Multilocus Genotyping Identifies a Highly Homogeneous Phytoplasma Lineage Associated with Sweet Cherry Virescence Disease in China and Its Carriage by an Erythroneurine Leafhopper. Crop Prot..

[50] White D.T., Blackall L.L., Scott P.T., Walsh K.B. (1998). Phylogenetic Positions of Phytoplasmas Associated with Dieback, Yellow Crinkle and Mosaic Diseases of Papaya, and Their Proposed Inclusion in “Candidatus Phytoplasma Australiense” and a New Taxon, “Candidatus Phytoplasma Australasia”. Int. J. Syst. Bacteriol..

[51] Zreik L., Carle P., Bové J.M., Garnier M. (1995). Characterization of the Mycoplasmalike Organism Associated with Witches’-Broom Disease of Lime and Proposition of a Candidatus Taxon for the Organism, “Candidatus Phytoplasma Aurantifolia”. Int. J. Syst. Bacteriol..

[52] Edgar R.C. (2004). MUSCLE: Multiple Sequence Alignment with High Accuracy and High Throughput. Nucleic Acids Res..

[53] Kumar S., Stecher G., Li M., Knyaz C., Tamura K. (2018). MEGA X: Molecular Evolutionary Genetics Analysis across Computing Platforms. Mol. Biol. Evol..

[54] Lanfear R., Calcott B., Ho S.Y.W., Guindon S. (2012). Partitionfinder: Combined Selection of Partitioning Schemes and Substitution Models for Phylogenetic Analyses. Mol. Biol. Evol..

[55] Lanfear R., Frandsen P.B., Wright A.M., Senfeld T., Calcott B. (2016). PartitionFinder 2: New Methods for Selecting Partitioned Models of Evolution for Molecular and Morphological Phylogenetic Analyses. Mol. Biol. Evol..

[56] Stamatakis A. (2014). RAxML Version 8: A Tool for Phylogenetic Analysis and Post-Analysis of Large Phylogenies. Bioinformatics.

[57] Edler D., Klein J., Antonelli A., Silvestro D. (2021). raxmlGUI 2.0: A Graphical Interface and Toolkit for Phylogenetic Analyses Using RAxML. Methods Ecol. Evol..

[58] Balbuena J.A., Míguez-Lozano R., Blasco-Costa I. (2013). PACo: A Novel Procrustes Application to Cophylogenetic Analysis. PLoS ONE.

[59] Balbuena J.A., Pérez-Escobar Ó.A., Llopis-Belenguer C., Blasco-Costa I. (2020). Random Tanglegram Partitions (Random TaPas): An Alexandrian Approach to the Cophylogenetic Gordian Knot. Syst. Biol..

[60] Hutchinson M.C., Cagua E.F., Balbuena J.A., Stouffer D.B., Poisot T. (2017). Paco: Implementing Procrustean Approach to Cophylogeny in R. Methods Ecol. Evol..

[61] Oksanen J., Blanchet F.G., Kindt R., Legendre P., Minchin P.R., O’Hara R.B., Simpson G.L., Solymos P., Stevens M.H.H., Wagner H. (2020). Vegan: Community Ecology Package. R Package Version 2.2-7. http://CRAN.Rproject.org/package=vegan.

[62] Legendre P., Desdevises Y., Bazin E. (2002). A Statistical Test for Host-Parasite Coevolution. Syst. Biol..

[63] Paradis E., Claude J., Strimmer K. (2004). APE: Analyses of Phylogenetics and Evolution in R Language. Bioinformatics.

[64] De Vienne D.M., Giraud T., Shykoff J.A. (2007). When Can Host Shifts Produce Congruent Host and Parasite Phylogenies? A Simulation Approach. J. Evol. Biol..

[65] R Core Team (2024). R: A Language and Environment for Statistical Computing.

[66] Conow C., Fielder D., Ovadia Y., Libeskind-Hadas R. (2010). Jane: A New Tool for the Cophylogeny Reconstruction Problem. Algorithms Mol. Biol..

[67] Ronquist F. (2002). TreeFitter.

[68] Charleston M.A., Page R.D.M. (2002). TreeMap 2. A Macintosh Program for Cophylogeny Mapping.

[69] Beaulieu J.M., O’Meara B.C., Donoghue M.J. (2013). Identifying Hidden Rate Changes in the Evolution of a Binary Morphological Character: The Evolution of Plant Habit in Campanulid Angiosperms. Syst. Biol..

[70] Trivellone V. Hemiptera-Phytoplasma-Plant Database Links. http://trivellone.speciesfile.org/.

[71] Fritz S.A., Purvis A. (2010). Selectivity in Mammalian Extinction Risk and Threat Types: A New Measure of Phylogenetic Signal Strength in Binary Traits. Conserv. Biol..

[72] Orme D., Freckleton R., Thomas G., Petzoldt T., Fritz S., Isaac N., Pearse W. (2025). Caper: Comparative Analyses of Phylogenetics and Evolution in R.

[73] Johnson K.P., Dietrich C.H., Friedrich F., Beutel R.G., Wipfler B., Peters R.S., Allen J.M., Petersen M., Donath A., Walden K.K.O. (2018). Phylogenomics and the Evolution of Hemipteroid Insects. Proc. Natl. Acad. Sci. USA.

[74] Wei W., Zhao Y. (2022). Phytoplasma Taxonomy: Nomenclature, Classification, and Identification. Biology.

[75] Merkle D., Middendorf M., Wieseke N. (2010). A Parameter-Adaptive Dynamic Programming Approach for Inferring Cophylogenies. BMC Bioinform..

[76] Llaberia-Robledillo M., Lucas-Lledó J.I., Pérez-Escobar O.A., Krasnov B.R., Balbuena J.A. (2023). Rtapas: An R Package to Assess Cophylogenetic Signal Between Two Evolutionary Histories. Syst. Biol..

[77] Agosta S.J. (2006). On Ecological Fitting, Plant-Insect Associations, Herbivore Host Shifts, and Host Plant Selection. Oikos.

[78] Kahn A.K., Almeida R.P.P. (2022). Phylogenetics of Historical Host Switches in a Bacterial Plant Pathogen. Appl. Environ. Microbiol..

[79] Navaud O., Barbacci A., Taylor A., Clarkson J.P., Raffaele S. (2018). Shifts in Diversification Rates and Host Jump Frequencies Shaped the Diversity of Host Range Among *Sclerotiniaceae* Fungal Plant Pathogens. Mol. Ecol..

[80] Wu F., Zheng Y., Wen X., Peng S., Deng K., Zhu Y., Lin X., Chen Y., Deng X., Cen Y. (2026). Mitochondrial Genomes of Psylloidea (Hemiptera): Candidate Broad-Range Primers for Long-Polymerase Chain Reaction, Genewise Variability, and Calibration-Sensitive Time-Calibrated Phylogeny. J. Insect Sci..

[81] Newbold T., Hudson L.N., Hill S.L.L., Contu S., Lysenko I., Senior R.A., Börger L., Bennett D.J., Choimes A., Collen B. (2015). Global Effects of Land Use on Local Terrestrial Biodiversity. Nature.

[82] Trivellone V., Wei W., Filippin L., Dietrich C.H. (2021). Screening Potential Insect Vectors in a Museum Biorepository Reveals Undiscovered Diversity of Plant Pathogens in Natural Areas. Ecol. Evol..

[83] Wei W., Trivellone V., Dietrich C.H., Zhao Y., Bottner-Parker K.D., Ivanauskas A. (2021). Identification of Phytoplasmas Representing Multiple New Genetic Lineages from Phloem-Feeding Leafhoppers Highlights the Diversity of Phytoplasmas and Their Potential Vectors. Pathogens.

[84] Trivellone V., Mackay A.J., Stone C.M., Dietrich C.H. (2026). Leveraging Existing Biodiversity and Zoonosis Monitoring Infrastructure for Integrative Plant Pathogen Surveillance in Natural Ecosystems. Insects.

[85] Cao Y., Dietrich C.H., Dmitriev D.A., Zou H., Xue Q., Zhang Y. (2026). Analyses of Expanded Anchored-Hybrid Phylogenomic Datasets Support Revisions to the Higher Classification of Leafhoppers (Hemiptera: Cicadellidae). Syst. Entomol..

