# Supplementary material for: First Cophylogenetic Reconstruction of Hemipteran Insect–Phytoplasma Associations Reveals Eco-Evolutionary Dynamics
Source: Insects. 2026 Sep 9;17(9):942. doi: 10.3390/insects17090942 (PMC13607781; doi:10.3390/insects17090942)
Supplement: Supplementary file 1 [file insects-17-00942-s001.zip › Figure S1.pdf]

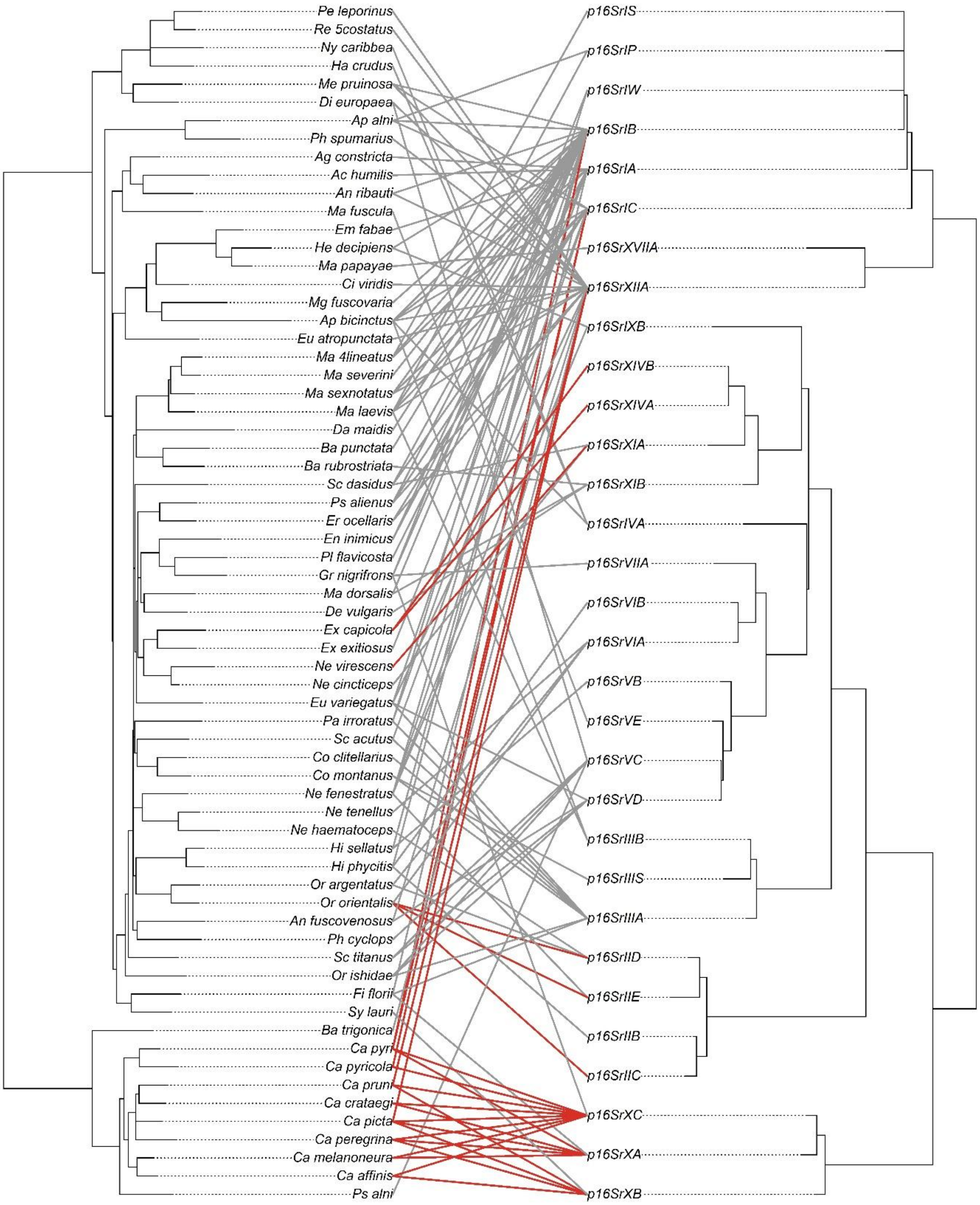

Figure S1. Tanglegram of the *full dataset* including experimentally confirmed vector-phytoplasma associations together with putative associations reported from phytoplasma detections in insects. Red lines highlight associations involving insect species which collectively accounted for the strongest cophylogenetic signal in both PACo and ParaFit

analyses. The topology summarizes the maximum-likelihood phylogenies reconstructed independently for insects and phytoplasmas.
